# A Novel Prognostic Scoring Model for Myelodysplastic Syndrome Patients With *SF3B1* Mutation

**DOI:** 10.3389/fonc.2022.905490

**Published:** 2022-06-27

**Authors:** Liya Ma, Bin Liang, Huixian Hu, Wenli Yang, Shengyun Lin, Lihong Cao, Kongfei Li, Yuemin Kuang, Lihong Shou, Weimei Jin, Jianping Lan, Xingnong Ye, Jing Le, Huyi Lei, Jiaping Fu, Ying Lin, Wenhua Jiang, Zhiying Zheng, Songfu Jiang, Lijuan Fu, Chuanyong Su, XiuFeng Yin, Lixia Liu, Jiayue Qin, Jie Jin, Shenxian Qian, Guifang Ouyang, Hongyan Tong

**Affiliations:** ^1^ Department of Hematology, The First Affiliated Hospital of Zhejiang University, Hangzhou, China; ^2^ Department of Hematology, The First Affiliated Hospital of Wenzhou University, Wenzhou, China; ^3^ Department of Hematology, Jinhua Central Hospital, Jinhua, China; ^4^ Department of Hematology, Zhejiang Provincial Hospital of Chinese Medicine, Hangzhou, China; ^5^ Department of Hematology, Shulan Hospital of Zhejiang Province, Hangzhou, China; ^6^ Department of Hematology, Ningbo Yinzhou People’s Hospital, Ningbo, China; ^7^ Department of Hematology, Jinhua People’s Hospital, Jinhua, China; ^8^ Department of Hematology, Huzhou Central Hospital, Huzhou, China; ^9^ Department of Hematology, Lishui People’s Hospital, Lishui, China; ^10^ Department of Hematology, Zhejiang Provincial People’s Hospital, Hangzhou, China; ^11^ Department of Hematology, The Fourth Affiliated Hospital of Zhejiang University, Yiwu, China; ^12^ Department of Hematology, Ningbo Lihuili Hospital, Ningbo, China; ^13^ Department of Hematology, The Affiliated Hospital of Shaoxing University of Arts and Sciences, Shaoxing, China; ^14^ Department of Hematology, Shaoxing People’s Hospital, Shaoxing, China; ^15^ Department of Hematology, The Second Affiliated Hospital of Wenzhou University, Wenzhou, China; ^16^ Department of Hematology, Taizhou First People’s Hospital, Taizhou, China; ^17^ Department of Hematology, Xinhua Hospital of Zhejiang Province, Hangzhou, China; ^18^ Department of Hematology, Tongde Hospital of Zhejiang Province, Hangzhou, China; ^19^ Department of Hematology, The Affiliated Shaoyifu Hospital of Zhejiang University, Hangzhou, China; ^20^ Department of Medical Affairs, Acornmed Biotechnology Co., Ltd., Tianjin, China; ^21^ Department of Hematology, Hangzhou First People’s Hospital, Hangzhou, China; ^22^ Department of Hematology, Ningbo First Hospital, Ningbo, China

**Keywords:** Myelodysplastic syndrome (MDS), *SF3B1* mutation, *RUNX1*, *EZH2*, Ras, prognostic scoring model

## Abstract

The outcomes of myelodysplastic syndrome (MDS) patients with *SF3B1* mutation, despite identified as a favorable prognostic biomarker, are variable. To comprehend the heterogeneity in clinical characteristics and outcomes, we reviewed 140 MDS patients with *SF3B1* mutation in Zhejiang province of China. Seventy-three (52.1%) patients diagnosed as MDS with ring sideroblasts (MDS-RS) following the 2016 World Health Organization (WHO) classification and 118 (84.3%) patients belonged to lower risk following the revised International Prognostic Scoring System (IPSS-R). Although clonal hematopoiesis-associated mutations containing *TET2*, *ASXL1* and *DNMT3A* were the most frequent co-mutant genes in these patients, *RUNX1, EZH2, NF1* and *KRAS/NRAS* mutations had significant effects on overall survival (OS). Based on that we developed a risk scoring model as IPSS-R×0.4+*RUNX1*×1.1+*EZH2*×0.6+*RAS*×0.9+*NF1*×1.6. Patients were categorized into two subgroups: low-risk (L-R, score <= 1.4) group and high risk (H-R, score > 1.4) group. The 3-year OS for the L-R and H-R groups was 91.88% (95% CI, 83.27%-100%) and 38.14% (95% CI, 24.08%-60.40%), respectively (P<0.001). This proposed model distinctly outperformed the widely used IPSS-R. In summary, we constructed and validated a personalized prediction model of MDS patients with *SF3B1* mutation that can better predict the survival of these patients.

## Introduction

In recent years, the molecular landscape of myelodysplastic syndrome (MDS) has been elucidated with the application of next-generation sequencing (NGS) (1). More than half of MDS patients carried splicing factor gene mutations, which have been indicated to be the most frequent molecular abnormality in this disease (2). Among these mutations, splicing factor 3 subunit 1 (*SF3B1*) is the most commonly mutated one. *SF3B1* locates at 2q33.1 with 25 exons and encodes a 1304 amino acid protein with a highly conserved nucleotide sequence, which is an important component of U2 snRNP (3). Base pairing between the U2 snRNP and the branch-point sequence is essential for pre-mRNA splicing (4). The SF3b/SF3a complex anchors the U2 snRNP to the pre-mRNA, and *SF3B1* is a crucial component of the activated spliceosome that helps the branch-point adenosine in place for nucleophilic attack from the 5’ splice site (3). *SF3B1* point mutations in MDS are limited to exons 14 through 16. The most common *SF3B1* mutation is an A-to-G transition that results in a lysine to glutamic acid substitution at amino acid position 700 (K700E) (5). *SF3B1* mutation alters U2 snRNP function by prompting alternative branch-point usage and induction of cryptic 3′ splice site selection, thereby forming aberrantly spliced mRNA transcripts subject to nonsense-mediated decay and downregulation of target transcripts and protein expression (6, 7).

About 20-28% of all MDS patients harbor *SF3B1* mutation (5, 8, 9) and a much higher occurrence rate of mutations has been detected in MDS with ring sideroblasts (MDS-RS), such as 64~83% in MDS-RS with single lineage dysplasia (MDS-RS-SLD) and 57~76% in MDS-RS with multiple lineage dysplasia (MDS-RS-MLD) (5, 9–11). Importantly, recent study shows that *SF3B1* mutation in MDS-RS can derive from the scarce hematopoietic stem cell compartment and is an initiating event in this disease (12). MDS patients with *SF3B1* mutation have higher platelet counts and lower bone marrow blast percentage in comparison to MDS patients with wild-type *SF3B1* (13). *SF3B1* mutations appear more commonly in lower risk MDS patients and are independent predictive factors of favorable prognosis in MDS (8). *SF3B1* mutations co-occur with mutations of genes involved in the regulation of DNA methylation, such as the methyltransferase *DNMT3A* and the methylcytosine dioxygenase *TET2* (14–17).

The International Prognostic Scoring System (IPSS) and the revised IPSS (IPSS-R) are the most widely applied models in clinical practice and clinical trial evaluation in MDS (18). IPSS or IPSS-R based upon the peripheral blood counts, percentage of bone marrow blasts and presence of cytogenetic abnormalities. However, neither IPSS nor IPSS-R incorporates gene mutations.

The International Working Group for the Prognosis of MDS (IWG-PM) has suggested that *SF3B1*mut MDS as a distinctive entity which has a favorable prognosis with <1% peripheral blood or <5% BM blasts, absence of del (5q), inv (3), abnormal 3q26, monosomy 7, or complex karyotype (CK) and *RUNX1* or *EZH2* mutations (19). This classification was mainly established on a specific gene mutation, association with ring sideroblasts and favorable prognosis. However, MDS with *SF3B1* mutation is a heterogeneous group since not all the patients had favorable survivals. The patients with excess blasts, poor cytogenetics and molecular genetic abnormalities had unfavorable survival (20).

In this study, we aimed to estimate the spectrum of *SF3B1* mutation-harboring MDS patients in Zhejiang Province of China, to analyze their clinical and laboratory characteristics and molecular landscape, and to explore the prognostic impacts of co-mutations. Furthermore, we constructed a prognostic model involving IPSS-R and selected gene mutations of MDS patients with *SF3B1* mutation to predict their outcomes.

## Patients and Methods

### Patients

**Figure 1:**
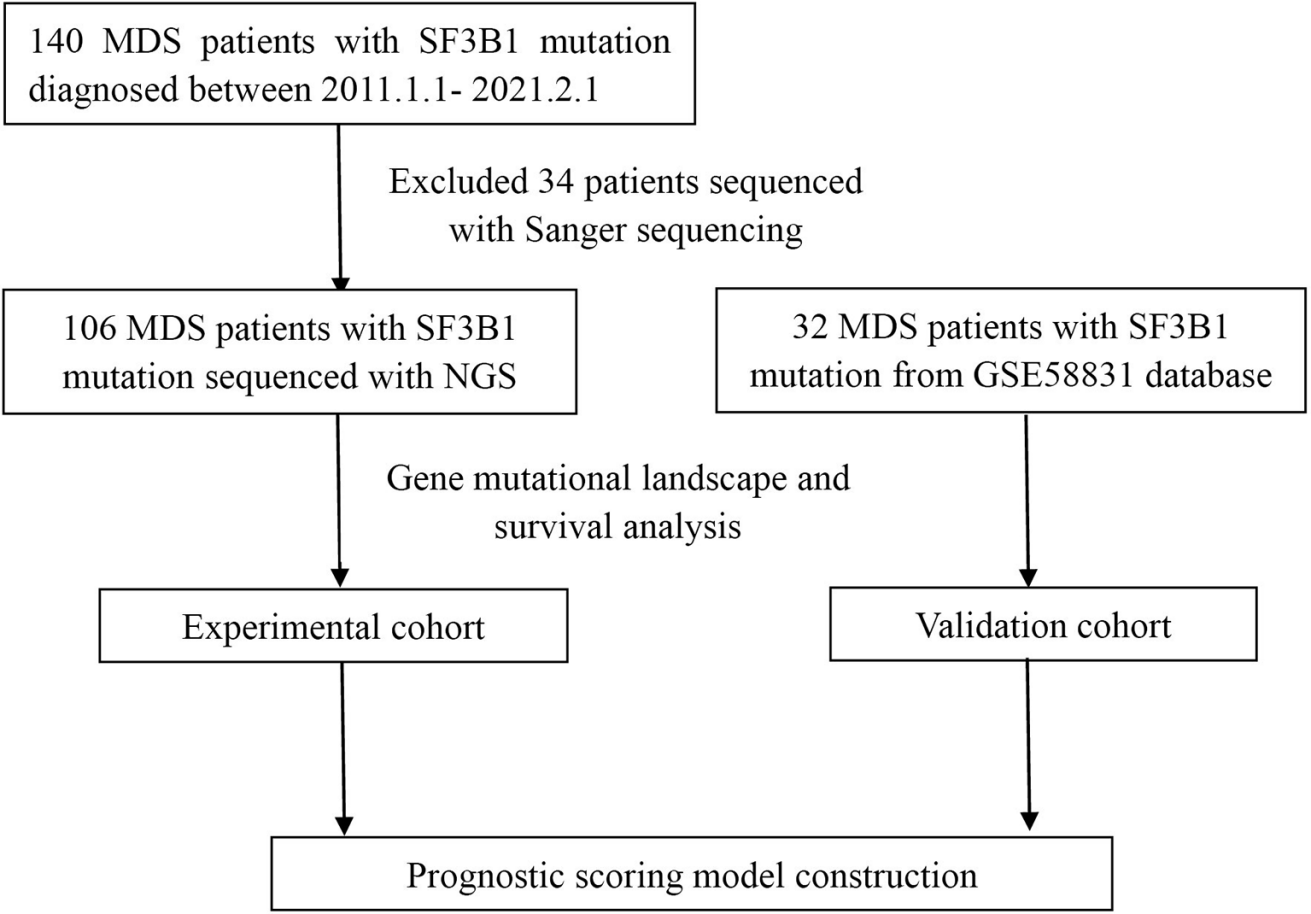
Flowchart of MDS patients with *SF3B1* mutation.

We reviewed the diagnosed cases of MDS with *SF3B1* mutation from January 1, 2011 through February 1, 2021 at the First Affiliated Hospital of Zhejiang University and other twenty-one hospitals in Zhejiang Province of China (**Figure 1**). Clinical, hematological, cytogenetic and molecular data were collected for all patients. Totally one hundred and forty patients were enrolled and classified according to 2016 WHO definition and classification of MDS (21). IPSS-R was used to evaluate the prognosis of each patient (18). This study was approved by the Ethics Committee of the First Affiliated Hospital of Zhejiang University consistent with Declaration of Helsinki.

### Cytogenetic Analysis

Bone marrow (BM) aspirates were cultured for 24 or 48h without mitogens and metaphase cells were prepared for analysis. Chromosomal analysis was performed following standard protocols and the results were reported in accordance with International System for Human Cytogenetic Nomenclature (ISCN) 2016 (22). At least 20 metaphase cells were tested if available. On the basis of IPSS-R, cytogenetic risk was categorized into five groups: very good risk, good risk, intermediate risk, high risk and very high risk (18).

### Gene Sequencing Analysis

Genomic DNA was extracted from mononuclear cells of BM samples at diagnosis of MDS. The Sanger sequencing was performed to detect gene mutations in 34 patients diagnosed from 2011 to 2014. NGS platforms covering 34~185 genes were performed to detect gene mutations in 106 patients diagnosed from 2015 to 2019 because NGS was widely applied since 2015 in Zhejiang Province. Multiplex libraries were sequenced using Illumina NovaSeq instrument. Burrows-Wheeler alignment (BWA, version 0.7.12) was used to align the trimmed reads. MarkDuplicates tool from Picard was performed to mark PCR duplicates. IndelRealigner and BaseRecalibrator from Genome Analysis Toolkit (GATK, version 3.8) were performed to realign and recalibrate the BWA data, respectively. Mutect2 was applied to call variants, including SNVs and InDels. ANNOVAR software was used for annotating all the variants including 1000G projects, COSMIC, PolyPhen and SIFT.

### Statistical Analysis

The SPSS (version 25) and R (version 3.6.3) software were used to conduct statistical analysis. Mann-Whitney U test was applied for continuous variables and chi-square test was applied for categorical variables. Overall survival (OS) was calculated as the period from the day of diagnosis to the day of death regardless any cause or last contact. OS curves were constructed by the Kaplan-Meier method and the differences in survival curves were compared by the log-rank test. Cox proportional hazard regression analysis was used to examine different independent prognostic factors for OS. The least Absolute Shrinkage and Selector Operation (LASSO) Cox regression model was used for variable selection and predictive prognostic model construction. A two-tailed P < 0.05 was deemed as statistically significant.

## Results

### Clinical Characteristics of MDS Patients With *SF3B1* Mutation

A total of 140 MDS patients from 22 hospitals in Zhejiang Province between January 2011 and February 2021 carried *SF3B1* mutation. The clinical characteristics of the MDS patients were listed in **Table 1**. The patients contained 83 men and 57 women, with a median age of 66 (range, 26-95) years. The median percent of BM blasts was 1.5% (0-19%). According to 2016 WHO sub-classifications, 53 patients (37.9%) diagnosed as MDS-RS-SLD; 20 patients (14.3%) diagnosed as MDS-RS-MLD; 11 patients (7.9%) as MDS-SLD; 27 patients (19.3%) as MDS-MLD; 12 (8.6%) patients as MDS-EB1; 12 (8.6%) patients as MDS-EB2; 1 (0.7%) patient as del(5q) syndrome; and 4 (2.9%) patients as MDS-unclassifiable (MDS-U). According to the cytogenetic risk stratification, only one patient (0.7%) categorized to the very good group, 117 patients (83.6%) to the good group, 15 patients (10.7%) to the intermediate group, 6 patients (4.3%) to the poor group and one patient (0.7%) to the very poor group. Following the IPSS-R, 6 (4.3%) patients were very low risk; 67 (47.9%) patients were low risk, 45 (32.1%) patients were intermediate risk, 14 (10.0%) patients were high risk and 8 (5.7%) patients were very high risk. With respect to treatment, 69 (49.3%) patients received erythroid stimulating agents (ESA) alone or combined with testosterone undecanoate and retinoic acid, 47 (33.8%) received supportive care and 24 (17.2%) received hypomethylating agents (HMAs) alone or combined with chemotherapy. Only 9 patients (5.4%) transformed to Acute myeloid leukemia (AML) in the course of disease and 38 patients (27.1%) died during follow-up. With a median follow-up of 21.77 months (range, 11.33-52.77), the median time to AML progressions was 13.15 months (range, 4.77-47.7).

**Table 1:** Clinical and laboratory characteristics of 140 MDS patients with *SF3B1* mutations.

Variables	Total (n=140)
Age, median (range)	66 (26-95)
Gender (male/female) Clinical characteristics	1.5 (83/57)
WBC (×10^9^/L), median (range)	3.0 (0.6-8.9)
ANC (×10^9^/L), median(range)	1.7 (0.2-6.9)
HB (g/L), median (range)	71.0 (29.0-124.0)
PLT (×10^9^/L), median (range)	150 (10-583)
BM blasts (%), median (range)	1.5 (0-19.0)
Ring sideroblasts (%), median (range)	7.5 (0-67.0)
2016 WHO categories, n (%)	
MDS-RS-SLD	53 (37.9)
MDS-RS-MLD	20 (14.3)
MDS-SLD	11 (7.9)
MDS-MLD	27 (19.3)
MDS-EB1	12 (8.6)
MDS-EB2	12 (8.6)
MDS-U	4 (2.9)
5q- syndrome	1 (0.7)
Cytogenetics (%)	
Very good	1 (0.7)
Good	117 (83.6)
Intermediate	15 (10.7)
Poor	6 (4.3)
Very poor	1 (0.7)
IPSS-R risk stratification, n (%)	
Very low	6 (4.3)
Low	67 (47.9)
Intermediate	45 (32.1)
High	14 (10.0)
Very high	8 (5.7)
AML transformation (%)	9 (6.4)
Death (%)	38 (27.1)

WBC, white blood cells; ANC, absolute neutrophil count; HB, Hemoglobin; PLT, Platelets; WHO, World Health Organization; MDS, myelodysplastic syndrome; RS, ring sideroblast; SLD, single lineage dysplasia; MLD, multilineage dysplasia; EB, excessive blasts; MDS-U, MDS unclassifiable; IPSS-R, the Revised International Prognostic Scoring System for MDS; AML, acute myeloid leukemia.

### Mutational Landscape of MDS Patients With *SF3B1* Mutation

**Figure 2:**
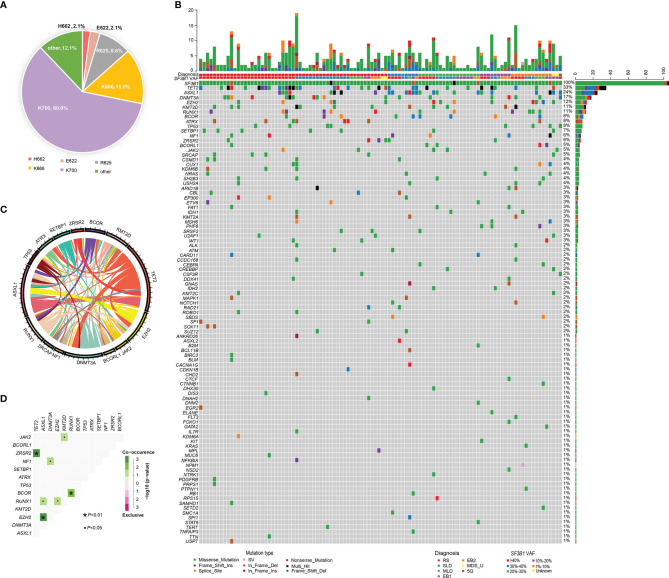
Genomic landscape of MDS patients with *SF3B1* mutation. **(A)** Distribution and proportion of *SF3B1* mutation sites in the 140 MDS patients. **(B)** Heatmap in 106 MDS patients with *SF3B1* mutation. Each row represents mutated gene; each column represents a patient; the right side of the graph annotates the frequency and number of the mutated gene; the upper histogram shows the number of gene mutations per patient; different colors below the graph represent different mutation patterns. **(C)** Circos diagram shows gene association in 106 MDS patients with *SF3B1* mutation, according to the relative frequency and pairwise co−occurrence of gene mutations on the basis of the mutated genes detected in ≥5 patients. **(D)** Diagram shows pairwise gene mutation correlations on the basis of the mutated genes detected in ≥5 patients, green color represents co-occurrence and pink color represents exclusivity.

BM aspirates from 106 MDS patients with *SF3B1* mutation underwent NGS analysis with 34~185 gene panels at the time of diagnosis. The median variant allele fraction (VAF) of *SF3B1* mutations was 38.0% (range, 1.2% to 50.1%). The most frequent *SF3B1* mutation site was K700E (n=84, 60.0%), followed by K666 (n=21, 15.0%), R625 (n=12, 8.6%), E622 (n=3, 2.1%), H662 (n=3, 2.1%) and others (n=17, 12.1%) (**Figure 2**). Ninety-eight mutant genes except *SF3B1* were detected in the 106 patients, and the mutational landscape is described in **Figure 2**. Only 23 patients (21.7%) had *SF3B1* mutation as the exclusive driver of MDS, while most patients (78.3%) had concomitant mutations. In order of decreasing frequency, commonly (> 5%) mutated genes included *TET2* (33.0%), *ASXL1* (23.6%), *DNMT3A* (16.0%), *EZH2* (12.3%), *RUNX1* (11.3%), *KMT2D* (11.3%), *BCOR* (8.5%), *ATRX* (7.5%), *TP53* (7.5%), *SETBP1* (6.6%), *NF1* (5.7%) and ZRSR2 (5.7%). Furthermore, the genes were categorized by function, revealing that chromatin modifying genes (23.4%), DNA methylation related genes (18.0%), signaling pathway genes (15.2%), transcription factor genes (10.1%) and histone methylation (9.8%) were most common (**Supplemental Figure 1**). The gene association analysis was performed for mutated genes detected in more than five patients, showing interesting coexistence and mutual exclusion relationships (**Figure 2**). Significant associations were discovered in paired genes, including *ZRSR2*-*TET2*, *EZH2*-*ASXL1*, *BCOR*-*RUNX1*, *RUNX1*-*EZH2*, *NF1*-*DNMT3A*, *JAK2*-*EZH2* and *RUNX1*-*ASXL1*, *JAK2*-*KMT2D* (P <0.001; P =0.001; P = 0.001; P = 0.018; P = 0.020; P = 0.022; P = 0.038; respectively) (**Figure 2**).

### Survival Analysis of MDS Patients With *SF3B1* Mutation

**Figure 3:**
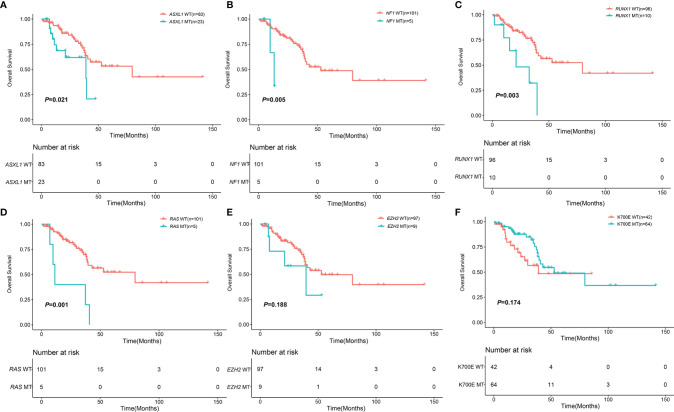
Impact of mutations on OS in 106 MDS patents with *SF3B1* mutation. **(A)** Kaplan-Meier curves comparing the OS of patients with *ASXL1* mutation (blue) compared with wild type (red) (39.03 months vs. 79.60 months, *P* = 0.021). **(B)** Kaplan-Meier curves comparing the OS of patients with *NF1* mutation (blue) compared with wild type (red) (13.17 months vs. 52.77 months, *P* = 0.005). **(C)** Kaplan-Meier curves comparing the OS of patients with *RUNX1* mutation (blue) compared with wild type (red) (21.03 months vs. 79.60 months, *P* = 0.003). **(D)** Kaplan-Meier curves comparing the OS of patients with *KRAS*/*NRAS* mutation (blue) compared with wild type (red) (11.33 months vs. 79.60 months, *P* = 0.001). **(E)** Kaplan-Meier curves comparing the OS of patients with *EZH2* mutation (blue) compared with wild type (red) (39.57 months vs. 52.77 months, *P* = 0.188). **(F)** Kaplan-Meier curves comparing the OS of patients with *SF3B1* K700E mutation (blue) compared with non *SF3B1* K700E (red) (52.77 months vs. 39.03 months, *P* = 0.174).

With a median follow-up of 22.22 months (range, 0.87-141.57), the 3-year OS of 106 MDS patients with *SF3B1* mutation was 68.30% (95%CI, 58.05-80.37%). The median number of co-mutant genes in these patients was 2 (0-17). The patients without *ASXL1* mutation had better survival than the patients with *ASXL1* mutation (79.60 months vs. 39.03 months, P=0.021) (**Figure 3**). Likewise, the patients without *NF1* mutation had longer survival than the patients with *NF1* mutation (48.76 months vs. 13.17 months, P=0.005) (**Figure 3**). *RUNX1* mutation was significantly associated with shorter survival (21.03 months vs. 79.60 months, P=0.003) (**Figure 3**). In addition, *KRAS/NRAS* mutation remarkably correlated with shorter survival (11.33 months vs. 79.60 months, P=0.001) (**Figure 3**). Nevertheless, *EZH2*, *TET2*, *DNMT3A*, *BCOR*, *KMT2D*, *ATRX*, *SETBP1*, *TP53* and *IDH1/2* had no impact on OS (**Figure 3** and **Supplemental Figure 2**). The patients with K700E had no survival advantage over the patients without K700E (52.77months vs. 39.03 months, P=0.174) (**Figure 3**). Furthermore, there was no difference in OS of patients with *SF3B1* K700E mutation compared with *SF3B1* K666N mutation (52.77 months vs. 29.63 months, P = 0.075) (**Supplemental Figure 2**).

### Prognostic Scoring Model of MDS Patients With *SF3B1* Mutations

To explore the prognostic factors of MDS patients with *SF3B1* mutation, we regarded MDS patients with *SF3B1* mutation in Zhejiang Province as the experimental cohort (n=106) and MDS patients with *SF3B1* mutation from GSE58831 database (23) as the validation cohort (n=32). A comparison of the basic characteristics of the patients in the experimental cohort and the validation cohort was listed in **Table 2**.

**Table 2:** Comparison of basic characteristics of patients in the experimental cohort and the verification cohort.

	experimental cohort (n=106)	validation cohort (n=32)	P value
Age, median (range)	66 (26-95)	69.5 (47-81)	0.479
gender (male/female)	1.4 (62/44)	1.4 (15/17)	0.246
WBC (×10^9^/L), median (range)	3.0 (0.9-8.9)	NA	NA
ANC (×10^9^/L), median(range)	1.6 (0.2-6.7)	2.68 (0.85-6.43)	<0.001
HB (g/L), median (range)	70.0 (29.0-120.0)	91.5 (69.0-131.0)	<0.001
PLT (×10^9^/L), median (range)	142 (10-583)	231 (35-604)	<0.001
BM blasts (%), median (range)	1.5 (0-19.0)	2.5 (0-15.0)	0.368
Ring sideroblasts (%), median (range)	6.0 (0-67.0)	NA	NA
2016 WHO categories, n (%)			0.037
MDS-RS-SLD	38 (35.8)	25 (78.1)	<0.001
MDS-RS-MLD	15 (14.2)		
MDS-SLD	10 (9.4)	NA	
MDS-MLD	19 (17.9)	2 (6.3)	
MDS-EB1	9 (8.5)	2 (6.3)	
MDS-EB2	12 (11.3)	1 (3.1)	
MDS-U	2 (1.9)	NA	
5q- syndrome	1 (0.9)	2 (6.3)	
Cytogenetics (%)			<0.001
Very good	1 (0.9)	0 (0)	
Good	89 (84.0)	23 (71.9)	
Intermediate	10 (9.4)	9 (28.1)	
Poor	5 (4.7)	0 (0)	
Very Poor	1 (0.9)	0 (0)	
IPSS-R risk stratification, n (%)			0.020
Very low	5 (4.7)	6 (18.8)	
Low	50 (47.2)	16 (50.0)	
Intermediate	30 (28.3)	10 (31.3)	
High	14 (13.2)	0 (0)	
Very high	7 (6.6)	0 (0)	

WBC, white blood cells; ANC, absolute neutrophil count; HB, Hemoglobin; PLT, Platelets; WHO, World Health Organization; MDS, myelodysplastic syndrome; RS, ring sideroblast; SLD, single lineage dysplasia; MLD, multilineage dysplasia; EB, excessive blasts; MDS-U, MDS unclassifiable; IPSS-R, the Revised International Prognostic Scoring System for MDS.

**Figure 4:**
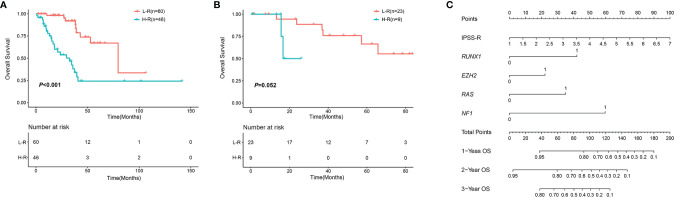
OS in different risk groups according to the novel scoring model. **(A)** OS in the experimental cohort. **(B)** OS in the validation cohort. **(C)** Nomogram for MDS patients with *SF3B1* mutation. An individual’s value is located on each variable axis, and a line is drawn upward to determine the points received for each variable. Corresponding points for each variable: IPSS-R scored as regular; *RUNX1* mutation scored 1; *EZH2* mutation scored 1; *KRAS/NRAS* mutation scored 1; *NF1* mutation scored 1; 0 for other conditions.

**Figure 5:**
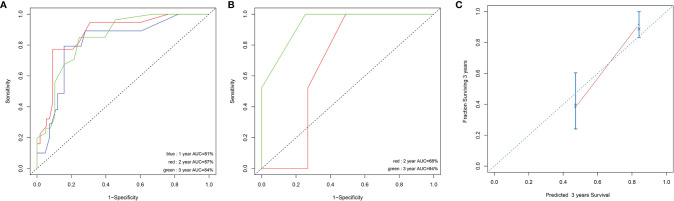
Discrimination ability with the use of the receiver operating characteristic curve in the experimental cohort **(A)** and validation cohort **(B)**. Calibration curves for predicting OS of MDS patients at 3 years in experimental cohort **(C)**. The sum of these points is located on the total point axis, and a line is drawn downward to the survival axis to determine the likelihood of 1,2, or 3-year OS.

Prognostic factors with P < 0.2 in the univariate analysis were performed to develop the prognostic scoring system and the results of univariate analysis were listed in **Supplemental Table 1**. Five variables were incorporated in the novel scoring model using LASSO Cox regression model. The risk scoring model was constructed including the weighted coefficients of these variables: IPSS-R×0.4+*RUNX1*×1.1+*EZH2*×0.6+*RAS*×0.9+*NF1*×1.6 (IPSS-R scored as regular; *RUNX1* mutation scored 1; *EZH2* mutation scored 1; *KRAS/NRAS* mutation scored 1; *NF1* mutation scored 1; 0 for other conditions). In the experimental cohort, 106 patients were classified into two subgroups on the basis of the risk score: low-risk (L-R, score <= 1.4, n = 60) and high risk (H-R, score > 1.4, n =46) groups. The 3-year OS for the L-R and H-R groups was 91.88% (95% CI, 83.27%-100%) and 38.14% (95% CI, 24.08%-60.40%), respectively (P<0.001) (**Figure 4**). In the validation cohort, the 3-year OS for the L-R and H-R groups was 88.54% (95% CI, 74.77%-100%) and 50.0% (95% CI, 18.77%-100%), respectively (P =0.052) (**Figure 4**). A prognostic nomogram that integrated all the five significantly independent variables from the LASSO Cox regression model was constructed (**Figure 4**). The nomogram was externally verified in the validation cohort. The predictive accuracy of the prognostic scoring model for OS in the experimental cohort evaluated with the C-index was 0.799 (95% CI, 0.764-0.834) which was higher than the C-index [0.765 (95% CI, 0.726-0.804)] of IPSS-R. Likewise, the C-index score [0.781 (95% CI, 0.715-0.847)] of the prognostic scoring model in the validation cohort was higher than the C-index [0.754 (95% CI, 0.687-0.821)] of IPSS-R. The calibration curves for predicting OS of patients after 3 years indicated an excellent conformity between the nomogram-predicted and actually observed values (**Figures 5A–C**).

## Discussion

In this study, using a NGS platform we explored the mutation profile in MDS patients with *SF3B1* mutations. We discovered that MDS patients with *SF3B1* mutation had many coexisting gene mutations, and the interactions were very complicated. Meanwhile, we found *RUNX1*, *EZH2, NF1* and *KRAS/NRAS* mutations had significant effects on prognosis. Based on these results, we proposed a scoring model combining both clinical features and gene mutations to predict outcomes in MDS patients with *SF3B1* mutation. Our proposed model distinctly surpassed the widely used IPSS-R. Our study might help to investigate the risk stratification and prognostic prediction, make reasonable decision and select appropriate therapies in *SF3B1* mutated MDS patients.

In accordance with previous reports (19, 24), more than half of MDS patients with *SF3B1* mutation were diagnosed as MDS-RS. The majority were categorized into good karyotype risk group and lower risk group according to IPSS-R. K700E was the most common mutation site of *SF3B1* in our study. But the patients with K700E had no survival advantage over the patients without K700E, which was inconsistent with the results from Rashmi KS, et al. showing that *SF3B1* mutated MDS with K700E had a remarkably better OS in contrast to non-K700E mutations (25).

As for the co-mutant genes, clonal hematopoiesis-associated mutations including *TET2*, *ASXL1* and *DNMT3A* were the most common co-mutant genes in the MDS patients with *SF3B1* mutations. However, *RUNX1*, *EZH2*, *NF1* and *KRAS/NRAS* mutations had significant effects on OS in our prognostic model, which coincided with the previous study (19) showing that *RUNX1*, *EZH2* and *NF1* mutations had significant effects on OS in *SF3B1*-mutant MDS patients within the IWG dataset.

The *RUNX1* transcription factor is a pivotal regulator of embryogenesis and hematopoiesis in vertebrates (23). *RUNX1* mutation is frequent in higher risk MDS such as MDS-MLD and MDS-EB. Furthermore, *RUNX1* mutation is correlated with poor clinical outcomes, particularly higher probability and shorter period for progression to AML (26, 27). The MDS patients with *RUNX1* mutation also have shorter OS (28, 29). Confirmed in our study, *RUNX1* mutation is an independent predictive factor of poor survival in MDS patients with *SF3B1* mutation (16, 30).

*EZH2*, located at chromosome 7q36, encodes for the catalytic subunit of the PRC2, which retains H3K27 methyltransferase activity. Inactivating mutation of *EZH2* is also found in MDS, which resulted in down-regulation of its expression (31–33). Deletion of Ezh2 in mice leads to MDS/MPN-like diseases, thus confirming the role of *EZH2* deficiency in disease development (31–34). In our study, although *EZH2* mutation had no impact on OS in univariate analysis (P=0.188), it showed significance in the multivariate analysis. Consistent with our study, *EZH2* mutation is also an independent predictive factor of poor survival in *SF3B1* mutated MDS (19).

This study had some limitations. First, not all the samples from *SF3B1* mutated patients were analyzed through NGS. Second, the number of patients in the verification cohort was relatively small. Therefore, a larger sample size study will be needed to verify our results.

In summary, we performed multi-gene sequencing and comprehensive prognostic analysis in MDS patients with *SF3B1* mutation. Our study pointed out the *SF3B1*mutation profile, revealed a novel scoring model combining both genetic and clinical outcomes that could stratify patients into two subgroups with distinct clinical outcomes, which play an important role in improving accuracy of prediction.

## Data Availability Statement

The original contributions presented in the study are publicly available. This data can be found here: https://db.cngb.org/search/project/CNP0003053/.

## Ethics Statement

The studies involving human participants were reviewed and approved by the Ethics Committee of the First Affiliated Hospital of Zhejiang University. The ethics committee waived the requirement of written informed consent for participation. Written informed consent was not obtained from the individual(s) for the publication of any potentially identifiable images or data included in this article.

## Authors Contributions

LM designed the research study and draft the manuscript. WY, BL, HH, GO, SQ, SL, LC, KL, YK, LS, WMJ, JPL, XNY, JL, HL, JF, LF, WHJ, ZZ, YL, LF, CS and XFY collected the data. LL and JQ performed the statistical analysis and drew diagrams. WY and SJ completed the follow-up. JJ and HT reviewed and revised the paper. All authors read and approved the final manuscript.

## Conflict of Interest

Authors LL and JQ are employed by Acornmed Biotechnology Co., Ltd.

The remaining authors declare that the research was conducted in the absence of any commercial or financial relationships that could be construed as a potential conflict of interest.

## Publisher’s Note

All claims expressed in this article are solely those of the authors and do not necessarily represent those of their affiliated organizations, or those of the publisher, the editors and the reviewers. Any product that may be evaluated in this article, or claim that may be made by its manufacturer, is not guaranteed or endorsed by the publisher.

## Supplementary Material

Supplementary Figure 1 Categories of the co-mutant genes by function.

Supplementary Figure 2 Impact of mutations on OS in 106 MDS patents with *SF3B1* mutation based on different mutated genes or mutation sites, including **(A)** Kaplan-Meier curves comparing the OS of patients with *TET2* mutation (blue) compared with wild type (red) (79.60 months vs. 40.67 months, *P* = 0.268). **(B)** Kaplan-Meier curves comparing the OS of patients with *DNMT3A* mutation (blue) compared with wild type (red) (42.93 months vs. 79.60 months, *P* = 0.253). **(C)** Kaplan-Meier curves comparing the OS of patients with *BCOR* mutation (blue) compared with wild type (red) (35.73 months vs. 52.77 months, *P* = 0.785). **(D)** Kaplan-Meier curves comparing the OS of patients with *KMT2D* mutation (blue) compared with wild type (red) (not reach vs. 42.93 months, *P* = 0.523). **(E)** Kaplan-Meier curves comparing the OS of patients with *ATRX* mutation (blue) compared with wild type (red) (not reach vs. 42.93 months, *P* = 0.535). **(F)** Kaplan-Meier curves comparing the OS of patients with *SETBP1* mutation (blue) compared with wild type (red) (not reach vs. 42.93 months, *P* = 0.572). **(G)** Kaplan-Meier curves comparing the OS of patients with *TP53* mutation (blue) compared with wild type (red) (not reach vs. 52.77 months, *P* = 0.971) **(H)** Kaplan-Meier curves comparing the OS of patients with *IDH1/2* mutation (blue) compared with wild type (red) (29.63 months vs. 52.77 months, *P* = 0.530). **(I)** Kaplan-Meier curves comparing the OS of patients with *SF3B1* K700E mutation (blue) compared with *SF3B1* K666N mutation (red) (52.77 months vs. 29.63 months, *P* = 0.075).

## Abbreviations

MDS, myelodysplastic syndrome; MDS-RS, MDS with ring sideroblasts; MDS-SLD, MDS with single lineage dysplasia; MDS- MLD, MDS with multiple lineage dysplasia; MDS-EB, MDS with excess blasts; MDS-U, MDS-unclassifiable; NGS, next-generation sequencing; *SF3B1*, splicing factor 3 subunit 1; *RUNX1*, Runt-related transcription factor 1; *EZH2*, Enhancer of zeste homolog 2; WHO, World Health Organization; IPSS-R, the revised International Prognostic Scoring System; IPSS, the International Prognostic Scoring System; IWG-PM, the International Working Group for the Prognosis of MDS; ISCN, International System for Human Cytogenetic Nomenclature; OS, Overall survival; LASSO, the least Absolute Shrinkage and Selector Operation; ESA, erythroid stimulating agents; HMAs, hypomethylating agents; AML, Acute myeloid leukemia; VAF, variant allele fraction.
