# Supplementary figures and images for: A Novel Prognostic Scoring Model for Myelodysplastic Syndrome Patients With SF3B1 Mutation
Source: Front Oncol. 2022 Jun 27;12:905490. doi: 10.3389/fonc.2022.905490 (PMC9271788; doi:10.3389/fonc.2022.905490)

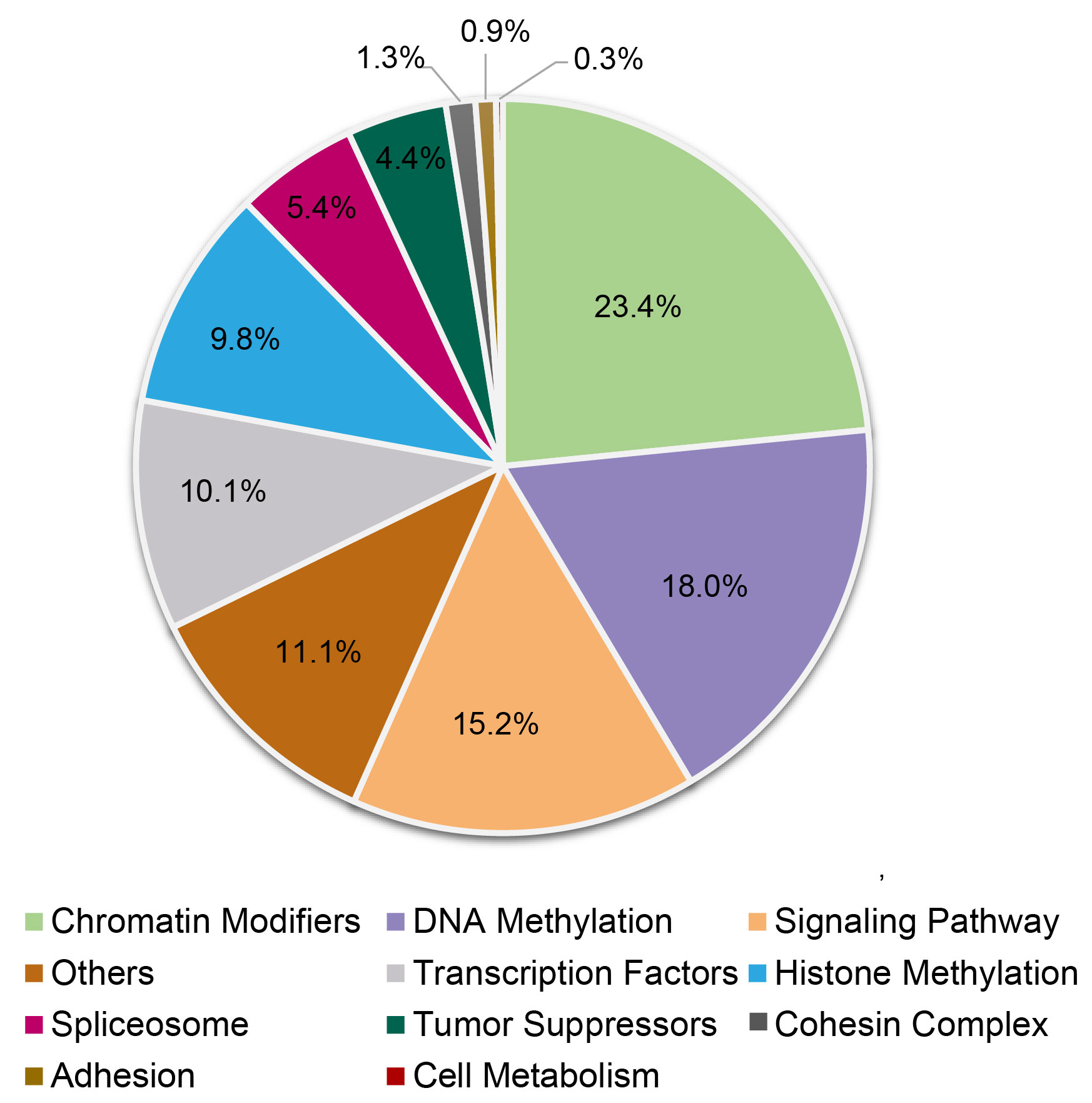

Supplement: Supplementary Figure 1 — Categories of the co-mutant genes by function. [file Image_1.jpeg]

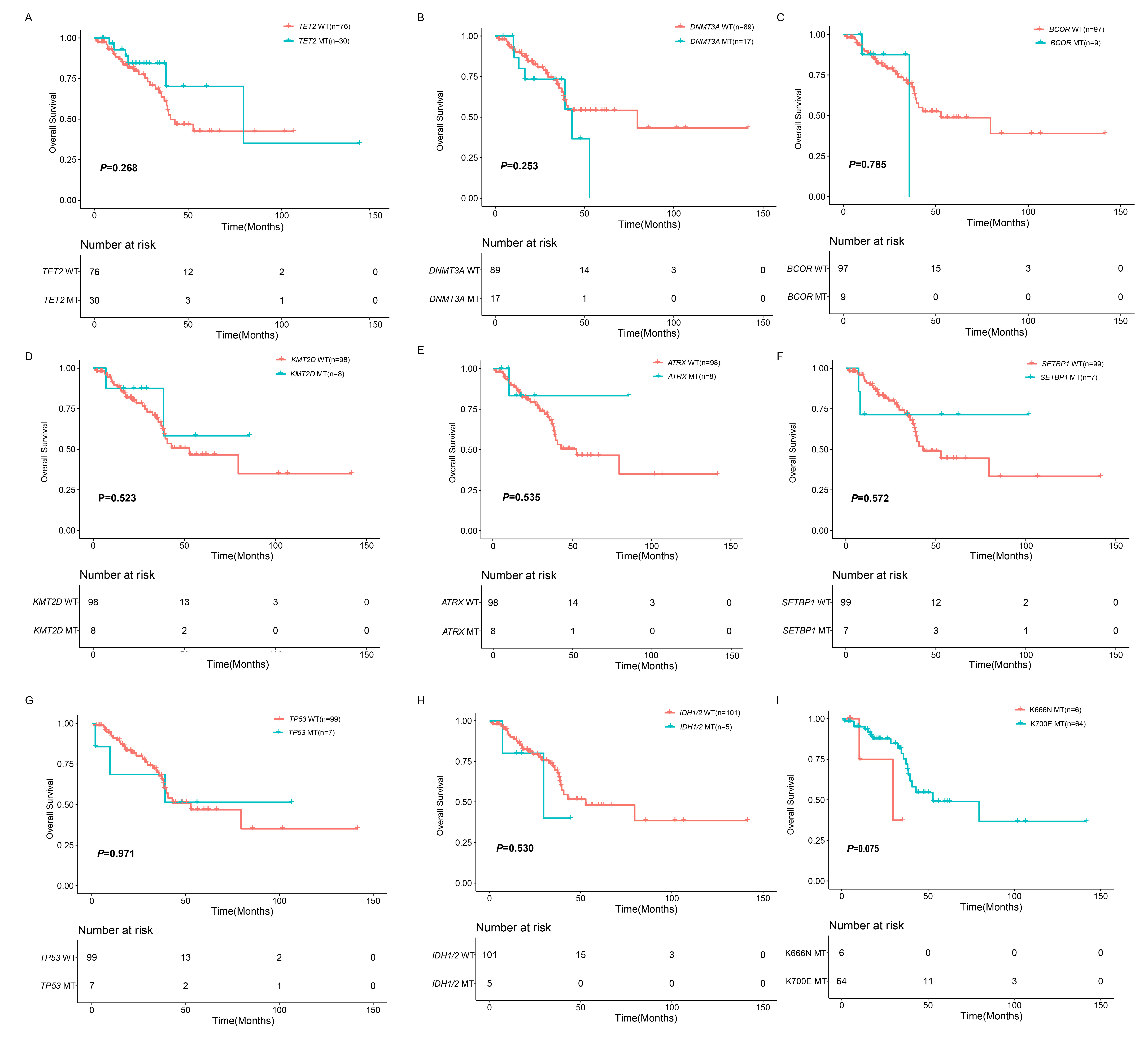

Supplement: Supplementary Figure 2 — Impact of mutations on OS in 106 MDS patents with SF3B1 mutation based on different mutated genes or mutation sites, including (A) Kaplan-Meier curves comparing the OS of patients with TET2 mutation (blue) compared with wild type (red) (79.60 months vs. 40.67 months, P = 0.268). (B) Kaplan-Meier curves comparing the OS of patients with DNMT3A mutation (blue) compared with wild type (red) (42.93 months vs. 79.60 months, P = 0.253). (C) Kaplan-Meier curves comparing the OS of patients with BCOR mutation (blue) compared with wild type (red) (35.73 months vs. 52.77 months, P = 0.785). (D) Kaplan-Meier curves comparing the OS of patients with KMT2D mutation (blue) compared with wild type (red) (not reach vs. 42.93 months, P = 0.523). (E) Kaplan-Meier curves comparing the OS of patients with ATRX mutation (blue) compared with wild type (red) (not reach vs. 42.93 months, P = 0.535). (F) Kaplan-Meier curves comparing the OS of patients with SETBP1 mutation (blue) compared with wild type (red) (not reach vs. 42.93 months, P = 0.572). (G) Kaplan-Meier curves comparing the OS of patients with TP53 mutation (blue) compared with wild type (red) (not reach vs. 52.77 months, P = 0.971) (H) Kaplan-Meier curves comparing the OS of patients with IDH1/2 mutation (blue) compared with wild type (red) (29.63 months vs. 52.77 months, P = 0.530). (I) Kaplan-Meier curves comparing the OS of patients with SF3B1 K700E mutation (blue) compared with SF3B1 K666N mutation (red) (52.77 months vs. 29.63 months, P = 0.075). [file Image_2.jpeg]
