# Supplementary material for: A Novel Prognostic Scoring Model for Myelodysplastic Syndrome Patients With SF3B1 Mutation
Source: Front Oncol. 2022 Jun 27;12:905490. doi: 10.3389/fonc.2022.905490 (PMC9271788; doi:10.3389/fonc.2022.905490)
Supplement: Supplementary file 3 [file Table_1.docx]

**Supplemental Table1**: Cox univariate analysis for overall survival in 106 MDS patients with *SF3B1* mutations

| Factors | HR (95%CI) | *P* value |
| --- | --- | --- |
| Age (≥60y vs <60y) | 0.872(0.441-1.726) | 0.692 |
| WBC (<3.0 vs ≥3.0*10^9^/L) | 0.803(0.406-1.589) | 0.528 |
| ANC (<1.5 vs ≥1.5*10^9^/L) | 0.636(0.317-1.274) | 0.187 |
| HB (<80 vs ≥80g/L) | 0.771(0.379-1.569) | 0.489 |
| PLT (<100 vs ≥100*10^9^/L) | 0.345(0.166-0.718) | 0.001 |
| BM Blast (≥5% vs <5%) | 4.595(1.71-12.349) | <0.001 |
| IPSS-R (＞3.5 vs≤3.5) | 4.816(2.276-10.188) | <0.001 |
| *RAS* (MT vs WT) | 4.533(0.759-27.060) | 0.001 |
| *RUNX1*(MT vs WT) | 3.493(0.829-14.723) | 0.003 |
| *NF1* (MT vs WT) | 5.919(0.218-161.085) | 0.005 |
| *ASXL1* (MT vs WT) | 2.369(0.877-6.395) | 0.021 |
| K700E vs K666N | 0.294(0.024-3.605) | 0.174 |
| *EZH2* (MT vs WT) | 1.987(0.497-7.935) | 0.188 |
| *DNMT3A* (MT vs WT) | 1.616(0.609-4.285) | 0.253 |
| *TET2* (MT vs WT) | 0.612(0.283-1.323) | 0.268 |
| *ZRSR2* (MT vs WT) | 2.127(0.124-36.365) | 0.658 |
| *U2AF1* (MT vs WT) | 1.736(0.277-10.887) | 0.437 |
| *KMT2D* (MT vs WT) | 0.631(0.195-2.046) | 0.523 |
| *ATRX* (MT vs WT) | 0.541(0.121-2.431) | 0.535 |
| *SETBP1* (MT vs WT) | 0.666(0.2-2.219) | 0.572 |
| *IDH1/2* (MT vs WT) | 1.572(0.272-9.081) | 0.530 |
| *SRSF2* (MT vs WT) | 1.584(0.133-18.865) | 0.646 |
| *BCOR* (MT vs WT) | 1.217(0.256-5.791) | 0.785 |
| *TP53* (MT vs WT) | 1.022(0.309-3.385) | 0.971 |

Abbreviations: WBC: white blood cells; ANC: absolute neutrophil count; HB: Hemoglobin; PLT: Platelets; BM: bone marrow; HR: Hazard Ratio; CI: confidence interval; MT: mutated; WT: wild-type.
